# Development of a marker panel for genotyping of domestic soybean cultivars for genes controlling the duration of vegetation and response to photoperiod

**DOI:** 10.18699/VJ21.087

**Published:** 2021-11

**Authors:** R.N. Perfil’ev, A.B. Shcherban, E.A. Salina

**Affiliations:** Institute of Cytology and Genetics of the Siberian Branch of the Russian Academy of Sciences, Novosibirsk, Russia; Institute of Cytology and Genetics of the Siberian Branch of the Russian Academy of Sciences, Novosibirsk, Russia; Institute of Cytology and Genetics of the Siberian Branch of the Russian Academy of Sciences, Novosibirsk, Russia

**Keywords:** photoperiod, flowering period, gene marker, allele-specific primers, nonsynonymous substitution, indel, cultivar, soybean, maturity group, фотопериод, срок цветения, маркер гена, аллель-специфичные праймеры, несинонимичная замена, индель, сорт, соя, группа спелости

## Abstract

Soybean, Glycine max L., is one of the most important agricultural crops grown in a wide range of latitude. In this regard, in soybean breeding, it is necessary to pay attention to the set of genes that control the transition to the f lowering stage, which will make it possible to adapt genotypes to local growing conditions as accurately as possible. The possibilities of soybean breeding for this trait have now signif icantly expanded due to identif ication of the main genes (E1–E4, GmFT2a, GmFT5a) that control the processes of f lowering and maturation in soybean, depending on the day length. The aim of this work was to develop a panel of markers for these genes, which could be used for a rapid and eff icient genotyping of domestic soybean cultivars and selection of plant material based on sensitivity to photoperiod and the duration of vegetation. Combinations of 10 primers, both previously developed and our own, were tested to identify different alleles of the E1–E4, GmFT2a, and GmFT5a genes using 10 soybean cultivars from different maturity groups. As a result, 5 combinations of dominant and recessive alleles for the E1–E4 genes were identif ied: (1) e1-nl(e1-as)/ e2-ns/e3-tr(e3-fs)/e4; (2) e1-as/e2-ns/e3-tr/E4; (3) e1-as/e2-ns/E3-Ha/e4; (4) E1/e2-ns/e3-tr/E4; (5) e1-nl/e2-ns/E3-Ha/E4. The studied cultivars contained the most common alleles of the GmFT2a and GmFT5a genes, with the exception of the ‘Cassidi’ cultivar having a rare dominant allele GmFT5a-H4. The degree of earliness of cultivars positively correlated with the number of recessive genes E1–E4, which is consistent with the data of foreign authors on different sets of cultivars from Japan and North China. Thus, the developed panel of markers can be successfully used in the selection of soybean for earliness and sensitivity to photoperiod.

## Abstract (ru)

Cоя (Glycine max L.) – одна из важнейших сельскохозяйственных культур, выращиваемая в большом диапазоне географической широты. В связи с этим в селекции сои необходимо обращать внимание на набор генов, контролирующих переход к фазе цветения, что позволит максимально точно адаптировать генотипы к локальным условиям произрастания. В настоящее время возможности селекции сои по данному признаку значительно расширились благодаря идентификации в ее геноме основных генов (E1–Е4, GmFT2a, GmFT5a), контролирующих процессы цветения и созревания в зависимости от длины дня. Целью нашей работы являлось создание панели маркеров к этим генам, которая может быть использована для быстрого и эффективного генотипирования отечественных сортов сои и отбора растительного материала по признакам чувствительности к длине дня и продолжительности вегетационного периода. Проведено тестирование 10 комбинаций праймеров (как ранее разработанных, так и собственных) для выявления различных аллельных состояний генов E1–Е4, GmFT2a и GmFT5a на выборке из 10 сортов сои из различных групп спелости. В итоге выявлено пять комбинаций доминантных и рецессивных аллелей по генам E1–E4: 1) е1-nl(e1-as)/e2-ns/е3-tr(e3-fs)/е4; 2) e1-as/e2-ns/е3-tr/Е4; 3) e1-as/e2-ns/Е3-На/е4; 4) E1/e2-ns/е3-tr/Е4; 5) е1-nl/e2-ns/Е3-На/Е4. Проанализированные сорта содержали наиболее распространенные аллели генов GmFT2a и GmFT5a, за исключением сорта Кассиди, у которого был обнаружен редкий доминантный аллель GmFT5a-H4. Степень скороспелости сортов положительно коррелировала с количеством рецессивных генов E1–Е4, что согласуется с данными зарубежных авторов, полученными на выборках сортов из Японии и Северного Китая. Таким образом, разработанная панель маркеров может успешно использоваться в селекции сои на скороспелость и чувствительность к фотопериоду.

## Introduction

The genus Glycine consists of two subgenera, Soja and Glycine. The first subgenus includes the species Glycine soja (2n = 4x = 40), or the Ussuri soybean – a wild annual plant from Southeast Asia and the cultivated species of soybean – Glycine max L. (2n = 4x = 40) (Vavilov, 1926; Zhukovsky, 1964).

Soybean is cultivated in many countries of the world for food, animal feed and technical purposes due to its unique nutritional properties, including a high protein content (30– 52 %). In terms of protein content, soybean surpasses all cultivated crops, in particular: wheat (9–26 %), rice (7 %), corn (10 %), etc., except for lupine. The value of soy protein is determined by the content of essential amino acids, the sum of which is 20 % of the total protein mass, and in wheat – 18 % (Gorissen et al., 2018). The degree of digestibility of soy protein has the highest index – 1, corresponding to proteins of milk, eggs, and casein and much higher than that of cereals (0.25–0.4) (Hoffman, Falvo, 2004).

Soybean was first cultivated in China 6000 BC. Then, as the main source for the production of vegetable protein and oil, soybean has spread to other countries of Southeast Asia: India, Korea, Japan, and Indonesia, where a variety of ways of eating it have been developed. Soybean appeared in Europe at the end of the 8th century. In Russia (the former USSR), soybean was brought to the Far East from China and this crop was introduced into production in the USSR in 1927.

In terms of the crop area in the world, soybean ranks first among leguminous crops. In 2019, it occupied 122 million hectares (https://www.kleffmann.com/). The world leaders in soybean production are Brazil and the United States. The cultivation area in these countries is 37 and 31 million hectares, respectively; average yield – 3.3 t/ha. According to the Federal State Statistics Service (Rosstat, https://rosstat.gov. ru/), in Russia in 2019, the total area under cultivated soybean was ~3 million hectares with yield – 1.0–2.0 t/ha. Five years later, the cultivation area of soybean in Russia has increased by 51 %. At the same time, the gross harvest increased by 1.6 times from 2.64 million tons in 2015 to 4.36 million tons in 2019.

The potential for increasing the yield of soybean in Russia is quite high and can be realized both by modernization of agrotechnical cultivation methods and through the development of new cultivars better adapted to the climatic conditions of specific regions (priority direction). The compatibility of the development phases with the optimum temperature for each phase plays an important role in plant adaptation. Soybean belongs to warm-season plants since the optimum temperature for the vegetative phase is +20…+25 °С and for seed germination – +12…+14 °С. Seedlings can withstand frosts down to –3 °C. During the period of flowering and pod maturity,the need for heat is greatest, with the optimum temperature during this period being +18…+20 °C.

Soybean is cultivated in a wide range of latitudes from 55° north to 35° south. However, the area of cultivation of each cultivar is limited to a very narrow range of latitudes and usually there is one cultivar per 1° of latitude (Agarkova et al., 2016). This is due to a strong reaction to the photoperiod. Soybean is a southern plant and it requires a short day to transition to flowering. In the long day environments in northern latitudes, the photoperiod-sensitive cultivars delay flowering and the pods do not have time to mature before the onset of frost in autumn. Reducing sensitivity to photoperiod allows the plant to start flowering earlier and reach maturity in the optimal period. On the other hand, in southern latitudes, in conditions of a short day and warm weather, soybean flowers too early and does not have time to form the vegetation mass necessary for the formation of a high yield.

Modulation of the maturity time, depending on the latitude of the area, is achieved by selecting an effective combination of gene alleles for this area, which are responsible for the photoperiodic reaction and the transition of the plant to flowering and maturation. At present, 11 major loci (E1–E11) affecting this trait have been identified in soybean (Jia et al., 2014; Tsubokura et al., 2014; Zhai et al., 2014, Samanfar et al., 2017; Wang et al., 2019). The function of genes E1–E4, which are directly involved in the regulation of flowering and maturity in various photoperiods, has been established in most detail (Xu et al., 2013). Combinations of the different alleles of these four genes account for 62–66 % variation in the length of the maturity time (Tsubokura et al., 2014). The E1 gene is a flowering repressor and encodes a transcription factor that contains the putative nuclear localization signal and the B3 DNA-binding domain (Watanabe et al., 2012; Xu et al., 2015). The E2 gene is an orthologue of the flowering regulator gene of the Arabidopsis GIGANTEA (Watanabe et al., 2011). The E3 and E4 genes encode phytochrome A: GmPHYA3 and GMPHYA2, respectively (Liu et al., 2008). Recessive alleles of genes E1–E4 are the result of mutations (frame shifts, nonsynonymous substitutions, deletions), leading to dysfunction of proteins, which gives insensitivity to photoperiod (Xu et al., 2013).

The soybean genome contains 12 GmFT genes homologous to the flowering activator FT (FLOWERING LOCUS T) of Arabidopsis (Kong et al., 2010; Wu et al., 2017). Of them, genes GmFT2a and GmFT4 were mapped as maturity genes E9 and E10, respectively (Zhao et al., 2016; Samanfar et al., 2017). The GmFT2a and GmFT5a genes have the strongest influence on the flowering time (Guo et al., 2015; Takeshima et al., 2016). Several signaling pathways for the regulation of soybean flowering depending on the photoperiod have been proposed, including the E1-specific regulatory pathway. According to this pathway, photoreceptors E3 and E4 provide photosensitivity and induce the expression of the E1 gene and its homologue E1L, which suppress the expression of GmFT5a and GmFT2a leading to a delay in flowering (Zhu et al., 2019).

Thus, the previous analysis of the main genes involved in the regulation of the maturity time in soybean made it possible to identify various dominant and recessive alleles of these genes, which cause different sensitivity to photoperiod, and to develop allele-specific markers for these genes. The aim of this work is to create a panel of molecular markers that can be used for fast and efficient genotyping of domestic soybean cultivars and selection of plant material in terms of sensitivity to day length and the duration of maturity.

## Materials and methods

As a material, we used 10 cultivars of soybean with different maturity time. Seeds of 4 cultivars were provided by Siberian Research Institute of Forages SFSCA RAS (Novosibirsk); 6 cultivars – by EFKO company (Alekseevka, Belgorod region; www.efcoforms.com). The names, genotypes and maturity time of the studied cultivars are presented in Table 1.

**Table 1.**
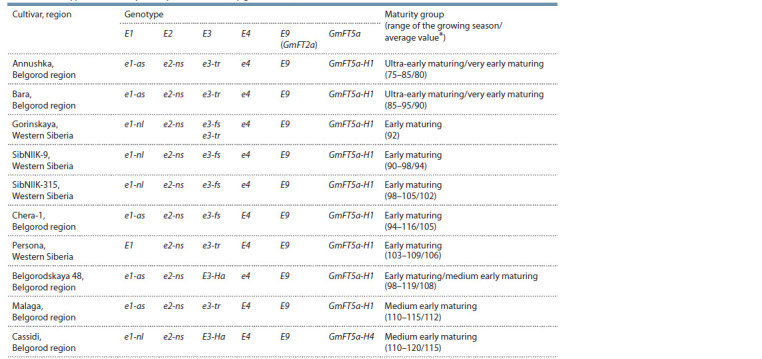
Genotypes of the analyzed soybean cultivars by genes E1–Е4, GmFT The duration of growing season on a long day was taken from the website of the State Register of Breeding Achievements (https://reestr.gossortrf.ru/). Maturity groups are given according to the classif ication generally accepted in Russia (Korsakov, 1973).

Total DNA was isolated from 4-day-old individual seedlings grown on wet filter paper in Petri dishes. DNA isolation was performed according to the method described by Kiseleva et al. (2016). The amount of DNA was determined using a spectrophotometer NanoDrop 2000 (Thermo Scientific, USA).

To identify the various alleles of the studied genes, we used allele-specific primers synthesized by “Biosset” company (Novosibirsk) (Table 2). PCR was performed in a 25-μl volume using a HS-Taq PCR kit (Biolabmix, Novosibirsk). The reaction mixture contained 50–100 ng of DNA, 1× PCR buffer, 2 mM MgCl2, 0.2 mM of each dNTP, 0.5 mM of each primer and 1 U HS-Taq DNA polymerase. PCR protocol: 5 min at 95 °C; 35–40 cycles (95 °С, 10 sec; 55–60 °С, 20 sec; 72 °С, 30–40 sec); 5 min at 72 °C. PCR products were separated by electrophoresis in 1 % agarose gel.

**Table 2.**
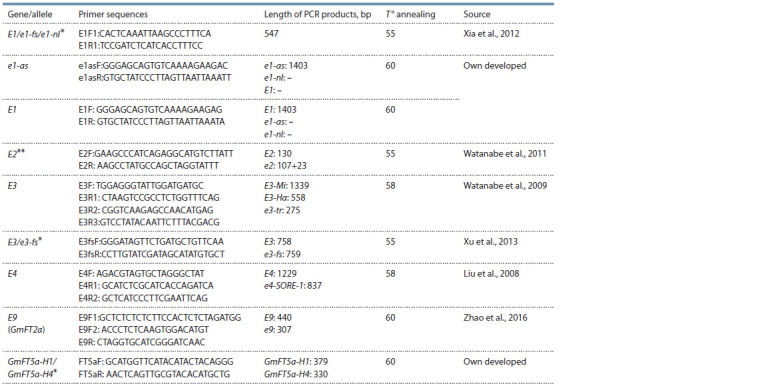
Primers used in the work * Combinations used for sequencing. ** CAPS marker with Dra I restriction enzyme.

To analyze the E2 gene, we used the CAPS marker described by Watanabe et al. (2011). The PCR product obtained using E2-specific primers was digested by restriction enzyme Dra I (SibEnzyme, Novosibirsk). We added 1 U of the enzyme to the PCR mixture and incubated it at 37 °C overnight. The restriction products were separated in 2 % agarose gel. The results of electrophoresis were visualized and photographed in UV using Gel Doc™ XR+ (BioRad, USA).

For sequencing, PCR products were isolated from the gel and purified using a diaGene kit for DNA elution from agarose gel (DiaM, Russia) according to manufacturer’s instruction. The sequencing of PCR products was carried out using a Bigdye terminator v3.1 cycle sequencing kit (Applied Biosystems, USA) and corresponding specific primers. Sequencing was performed at the SB RAS Genomics Core Facility using an automatic capillary analyzer ABI PRISM 310 Genetic Analyzer (Applied Biosystems, USA).

Comparison of the obtained sequences with those available from the NCBI database was performed using the BLASTN program (https://blast.ncbi.nlm.nih.gov/). Multiple alignment of DNA sequences was performed using the CLUSTAL Omega software (https://www.ebi.ac.uk/Tools/msa/clustalo/).

## Results

Previously, a number of studies carried out a detailed analysis of the structural organization of genes that determine the maturity time in soybean, including E-genes, as well as GmFT family genes (Liu et al., 2008; Xu et al., 2013; Jiang et al., 2014, 2019; Tsubokura et al., 2014). Molecular markers (PCR, CAPS markers) have been developed to identify different alleles of these genes, including the dominant alleles E1–E4 for photoperiod sensitive plants and recessive alleles that cause insensitivity to the photoperiod and reduce the maturity time. In this work, we tested these markers on a set of soybean cultivars approved for use in Russia to create a panel of molecular markers. This panel will allow for accelerated screening of cultivars based on sensitivity to photoperiod and genotyping for all the indicated genes.

**Fig. 1.**
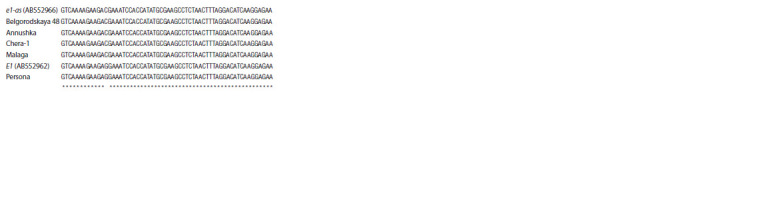
Multiple alignment of the E1 gene region containing an SNP characteristic of the recessive allele е1-as. E1 and е1-as – G. max E1 gene sequences of the ‘Harosoy’ cultivar (AB552962 and AB552966, respectively).

**Fig. 2.**
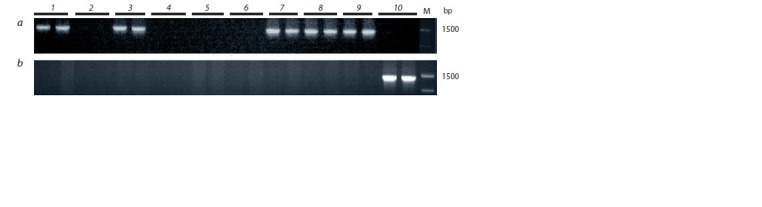
Results of PCR obtained using primers e1-asF/e1-asR (a) and E1F/E1R (b). Hereinafter: 1 – Annushka, 2 – Cassidi, 3 – Belgorodskaya 48, 4 – SibNIIK-9, 5 – SibNIIK-315, 6 – Gorinskaya, 7 – Chera-1, 8 – Bara, 9 – Malaga, 10 – Persona; M – “100+ bp DNA ladder”. Two individual plants were analyzed for each cultivar.

To analyze the E1 gene, we initially used a combination of primers E1F1/E1R1 common for dominant and recessive alleles and flanking a region of the coding sequence (see Table 2). This region contains SNPs specific for two common E1 recessive alleles: e1-fs and e1-as (Xia et al., 2012). As a result of PCR, a major 547 bp product was detected in 6 cultivars, while no PCR product was detected in the other 4 cultivars (result not shown). Then, we analyzed the nucleotide sequence of the obtained PCR product in 6 cultivars. Sequencing showed the presence of the e1-as allele in 5 cultivars and the E1 allele in the ‘Persona’ cultivar. The recessive allele e1-as is characterized by a nucleotide substitution G→C in comparison with the dominant allele E1 (Fig. 1). Based on the known sequences of the E1 gene from the databases, we developed the allele-specific primers e1asF/ e1asR, which allow us to identify the e1-as allele by the presence of a PCR product of 1403 bp (see Table 2). Figure 2, a shows the result of PCR with these primers. The next pair of primers (E1F/E1R) for the same region of the gene, specific for the dominant allele E1, gave an amplification only in the ‘Persona’ cultivar, which can be used as a control of E1 (see Fig. 2, b). The absence of PCR products with all primers to different regions of the E1 gene in cultivars ‘Cassidi’, ‘SibNIIK-9’, ‘SibNIIK-315’, ‘Gorinskaya’ can be explained by gene deletion, and this indicates the presence of the e1-nl allele, established by Xia et al. (2012).

**Fig. 3.**
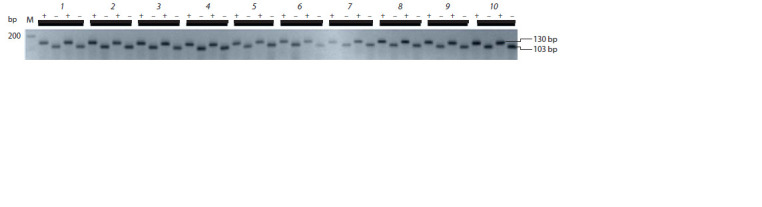
Electrophoregram of СAPS marker of the Е2 gene. Each plant sample is represented by a PCR product before (+) and after (–) restriction digestion.

We genotyped the E2 gene in cultivars using CAPS marker (see Table 2). The 130 bp PCR product of the dominant allele is not digested by endonuclease Dra I. The recessive allele e2 has a Dra I restriction site due to the A→T nucleotide substitution. The hydrolysis of the PCR product produces two DNA fragments 27 and 103 bp long. Figure 3 shows the presence of the recessive allele e2 in all studied cultivars.

**Fig. 4.**
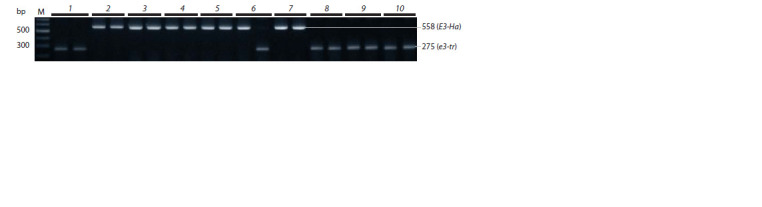
Electrophoregram of PCR products of the E3 gene. The products of 558 and 275 bp long correspond to the dominant E3-Ha and recessive e3-tr alleles, respectively

The E3 gene has the most common recessive allele e3-tr, which is characterized by a deletion of 13 kb after the third exon (Watanabe et al., 2009). The dominant alleles E3-Mi and E3-Ha have the same effect on the phenotype, but the last allele is distinguished by the insertion of a retrotransposon into the third intron. A molecular marker for this gene allows the simultaneous identification of both the dominant and recessive allele of the E3 gene (see Table 2). This marker revealed a 275 bp product characteristic of the recessive allele in the cultivars ‘Annushka’, ‘Bara’, ‘Persona’ and ‘Malaga’ and in one plant of the ‘Gorinskaya’ cultivar (Fig. 4). The rest of the samples had a PCR product corresponding to the dominant allele E3-Hа (see Fig. 4).

**Fig. 5.**
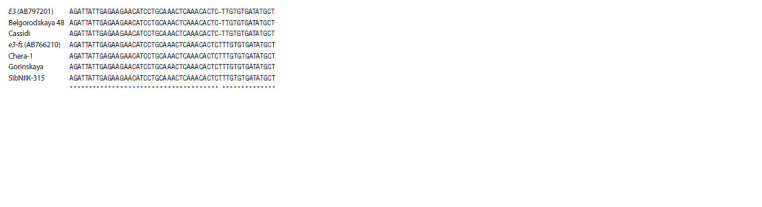
Multiple alignment of the f irst exon region of the E3 gene containing the insertion of T, leading to a frameshift mutation. The reference sequences of E3 and e3-fs alleles: AB797201 and AB766210, respectively.

In addition to the 13 kb deletion for E3, other mutations lead to the formation of recessive alleles. Among them, the most common allele is e3-fs with the insertion of a T nucleotide in the first exon, leading to a frame-shift and the formation of a non-functional protein (Xu et al., 2013). We checked this mutation in all cultivars with E3-Ha alleles (see above) by sequencing a 759/758 bp PCR product obtained with primers E3fsF/E3fsR (see Table 2, PCR result not presented). It turned out that cultivars ‘SibNIIK-9’, ‘SibNIIK-315’, ‘Gorinskaya’, ‘Chera-1’ are carriers of the allele e3-fs, and cultivars ‘Kassidi’, ‘Belgorodskaya 48’ have a sequence corresponding to the dominant allele E3-Hа (Fig. 5).

**Fig. 6.**
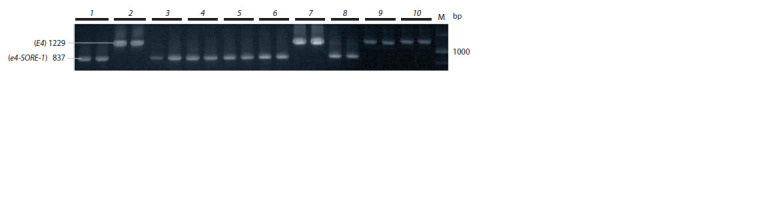
Electrophoregram of PCR products of the E4 gene. The products 1229 and 837 bp long correspond to the dominant E4 and recessive e4-SORE-1 alleles, respectively.

There are several recessive alleles of the E4 gene; the most common allele is e4-SORE-1, the result of the insertion of a 6,238 bp Ty1/copia-retrotransposon in the first exon (Liu et al., 2008). The molecular marker for this gene allows to identify simultaneously the dominant and recessive E4 alleles by the presence of PCR products 1229 bp and 837 bp long, respectively (see Table 2). Using this marker, we identified the dominant allele in cultivars ‘Cassidi’, ‘Chera-1’, ‘Malaga’ and ‘Persona’, while the other cultivars have a recessive allele (Fig. 6).

**Fig. 7.**
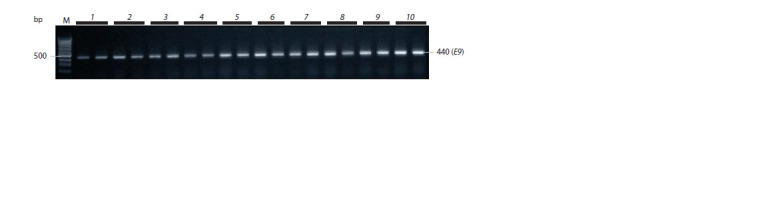
Electrophoregram of PCR product of the GmFT2a gene. The products of 379 and 330 bp long correspond to the recessive GmFT5a-H1 and dominant GmFT5a-H4 alleles, respectively.

Previously, molecular markers were developed for the flowering activator genes: GmFT2a, or the E9 gene (Zhao et al., 2016) and GmFT5a (Takeshima et al., 2016). The recessive allele e9 delays flowering due to lower gene expression caused by the insertion of the SORE-1 retrotransposon into the first intron (Zhao et al., 2016). The marker (see Table 2) allows determining the dominant and recessive allele GmFT2a, by the presence of PCR products 440 and 307 bp long, respectively. Using this marker, we identified a 440 bp PCR product characteristic of the dominant allele GmFT2a in all analyzed samples (Fig. 7).

**Fig. 8.**
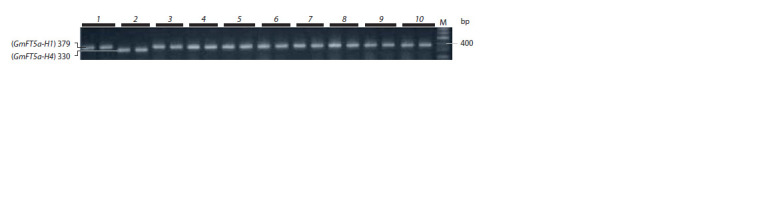
Electrophoregram of PCR products of the GmFT5a gene obtained with primers GmFT5af2/r2. The products of 379 and 330 bp long correspond to the recessive GmFT5a-H1 and dominant GmFT5a-H4 alleles, respectively.

The GmFT5a gene has a dominant allele, GmFT5a-H4, which reduces the maturity time and differs from the recessive allele by a 49 bp deletion in 3′-UTR (Takeshima et al., 2016; Jiang et al., 2019). To identify both GmFT5a alleles, we used a combination of primers FT5aF/FT5aR flanking the deletion site (see Table 2). A 330 bp PCR product corresponding to the dominant allele was detected in only one cultivar – ‘Cassidi’; the other cultivars had a 379 bp PCR product corresponding to the recessive allele (Fig. 8). We carried out sequencing of the PCR product in cultivars ‘Сassidi’ and ‘Belgorodskaya 48’ in order to search for the presence of different GmFT5a alleles. According to the sequencing result, the ‘Cassidi’ cultivar contained the GmFT5a-H4 allele (result not shown).

## Discussion

The high adaptation potential of soybean makes it possible to cultivate it outside the primary cultivation area – in a wide range of climatic conditions, including high-latitude regions with a temperate climate (Jia et al., 2014; Jiang et al., 2014). Soybean adaptation is achieved by the interaction of alleles of genes that control the date of flowering and maturity, depending on the length of the photoperiod (Saindon et al., 1989; Watanabe et al., 2012).

The maturity time of soybeans is 75 to 170 days. Depending on the maturity time, soybean cultivars are subdivided into: ultra-early maturing – less than 80 days; very early maturing – 81–90 days; early maturing – 91–110 days; medium early maturing – 111–120 days; medium maturing – 120–130 days; medium late maturing – 131–150 days; late maturing – 151–160 days; very late maturing – 161–170 days (Korsakov, 1973). In Russia, soybean is cultivated in the Far East, in the Central, Southern and Siberian regions. Each growing region is characterized by specific conditions of the climate; therefore,it becomes necessary to select cultivars specifically adapted to a particular region using effective methods of markerassisted selection. To demonstrate this possibility and create a working panel of DNA markers, we tested the previously developed combinations of primers for the main genes of photoperiod response: E1–E4 and flowering activators GmFT (Takeshima et al., 2016; Wu et al., 2017). For this purpose, we used a set of 10 cultivars, differing in times of maturity: from the ultra-early maturing cultivar ‘Annushka’ to the medium early maturing cultivar ‘Сassidi’ (average maturity time – 80 and 115 days, respectively). The established genotypes of these cultivars for all studied genes are presented in Table 1. In total, 5 combinations of alleles for the E1–E4 genes were identified: (1) е1-nl(e1-as)/e2-ns/е3-tr(e3-fs)/е4; (2) e1-as/ e2-ns/е3-tr/Е4; (3) e1-as/e2-ns/Е3-На/е4; (4) E1/e2-ns/ е3-tr/Е4; (5) е1-nl/e2-ns/Е3-На/Е4. 

All analyzed cultivars contained the most common, dominant and recessive alleles of the GmFT2a and GmFT5a genes, with the exception of the ‘Cassidi’ cultivar, which had a rare dominant allele GmFT5a-H4. The first combination E1–E4 was found in two ultra-early-maturing cultivars and three early-maturing cultivars close to them in terms of maturity time. This genotype is characterized by the presence of recessive alleles for each of the Е1–Е4 genes. The second combination with one dominant E4 gene is present in cultivars ‘Chera-1’ and ‘Malaga’ (maturity time: 105 and 112 days, respectively). The third combination with one dominant E3-Ha gene was found in the ‘Belgorodskaya 48’ cultivar (108 days). The fourth combination includes the dominant genes E1 and E4, found in the ‘Persona’ cultivar with a maturity time of 106 days. The medium early maturing cultivar ‘Сassidi’ contains the fifth combination with two dominant genes E3-Hа and E4 and has the longest maturity time in this sample of cultivars. This cultivar has the GmFT5a-H4 allele, which, according to Jiang et al. (2019), may influence the length of the maturity time. We have shown the predominant association of the genotype containing the recessive alleles of the E1–E4 genes with a group of ultra-early maturing and very early maturing cultivars, while cultivars with a later maturity time have one or two dominant alleles for the E1, E3, or E4 genes (see Table 1).

The established genotypes with a predominance of recessive alleles for the main genes of the photoperiod are typical for most cultivars from the northern regions of China (Jiang et al., 2014) and Japan (Xu et al., 2013). Thus, in the first work, it was found that the sensitivity to the photoperiod and the maturity time decrease with the accumulation of recessive alleles E1–E4. The cultivars with the genotype e1/e2/e3/e4 have the least sensitivity to photoperiod and are common in the northern latitudes of China. These cultivars belong to the MG000 maturity group of very early cultivars according to the international classification and correspond to ultra-early maturing and very early maturing cultivars according to our domestic classification. The MG00 and MG0 maturity groups of early and medium early cultivars have genotypes with one or two dominant genes, mainly E3 and E4 on the background of recessive alleles e1 and e2. These maturity groups have a maturity time of 91–110 and 111–120 days, respectively, which corresponds to our early and medium early maturing cultivars. Finally, MGI–MGIV maturity groups usually have genotypes with three or four dominant alleles: E1/e2/E3/E4, e1/E2/E3/E4, or E1/E2/E3/E4. These genotypes are common in the middle and southern regions of China, whose climatic conditions favor later maturation (Jiang et al., 2014). Thus, the analyzed cultivars have a maturity group MG000–MG0 and a genotype for genes E1–E4 similar to varieties from the northern regions of Southeast Asia, which are closest to the territory of the Far East – the region of primary soybean cultivation in our country. Soybean germplasm from this region has spread to the Southwestern part of Russia, Siberia and other regions.

Alleles E1–E4 have a different effect on sensitivity to photoperiod and maturity. Previous research shows that the E1 and E2 genes have a greater influence on the development prior to flowering. The loci E3 and E4 affect not only the previous, but also the subsequent phases of flowering and maturation (Xu et al., 2013; Jiang et al., 2014). Consequently, the last loci are more important in breeding for productivity. Of these genes, the E4 gene has the greatest effect on light sensitivity, the recessive form of which is quite widespread in northern latitudes, which is also confirmed by our data. Of the first two genes, the E1 gene presumably plays a key role in photoperiod- induced flowering (Xia et al., 2012). This is confirmed by the data of comparing the genotypes E1/e2/E3/E4 and e1/E2/E3/E4, which showed a more significant decrease in photoperiod response in the genotype with e1 (Jiang et al., 2014). Almost all cultivars studied by us, with the exception of the ‘Persona’ cultivar, contain non-functional alleles e1-as and e1-nl, which, apparently, make the main contribution to the shortening of the maturity time. The recessive allele e2 was found in all studied cultivars. Our result is consistent with the data from the Amur region, which showed the presence of the dominant allele E2 in only one cultivar out of 18 (Jia et al., 2014).

The genes of the GmFT family are flowering activators, and their transcription negatively correlates with the expression of the flowering repressor E1 (Xia et al., 2012). The most important genes of this family are genes GmFT2a and GmFT5a (Takeshima et al., 2016). Despite the fact that the GmFT2a gene showed different transcriptional profiles under different environmental conditions and in individual cultivars differing in sensitivity to photoperiod, nevertheless, its polymorphism was not associated with the maturity time (Jiang et al., 2013). In some cultivars, the insertion of the SORE-1 retrotransposon in the first intron of GmFT2a was identified, which suppressed the transcription of this gene and led to a delay in flowering (Zhao et al., 2016). Using the marker flanking the insertion (see Table 2), we established the intact form of the GmFT2a gene in all analyzed cultivars.

A 49 bp deletion in the 3′-UTR of the GmFT5a gene was found in a number of foreign cultivars of the MG000 and MG00 maturity groups (these groups also include the cultivars we analyzed), which reduces the flowering time relative to cultivars with a recessive allele of the gene (Takeshima et al., 2016; Jiang et al., 2019). We developed primers that amplify the site of the deletion, and using PCR and subsequent sequencing of the PCR product we showed the presence of this deletion in the ‘Cassidi’ cultivar (see Fig. 8). In addition to the indicated dominant allele GmFT5a, potentially shortening the flowering time, this cultivar contains two dominant alleles E3 and E4, which can have the opposite effect on the maturity time. However, the mechanism of interaction of these genes and their combined effect on the maturity time is yet to be clarified.

## Conclusion

In this work, using the material of soybean cultivars cultivated in Russia in the regions of Western Siberia and Belgorod region, we for the first time tested molecular markers for various alleles of the E1–E4, GmFT genes, which are responsible for sensitivity to photoperiod and the maturity time. Cultivars from these regions have a shorter maturity time and low sensitivity to photoperiod. These features correlate with the number of recessive alleles of the E1–E4 genes, so the cultivars with the shortest maturity time (ultra-early maturing) predominantly have the e1-nl(e1-as)/e2-ns/e3-tr(e3-fs)/e4 genotype. The cultivars with a later maturity (early maturing and medium early maturing) have a genotype with one or two dominant alleles, mainly for the E3 and E4 genes. Our result of genotyping 10 soybean cultivars is consistent with the data of foreign authors obtained on a wide set of cultivars from the geographical regions of Japan and North China, close in climatic conditions to the Far East – the region of primary soybean cultivation in our country. Thus, the tested set of molecular markers can be used for breeding the domestic soybean cultivars based on sensitivity to photoperiod and maturity time, on which the productivity of soybean largely depends, especially in a temperate climate atypical for its cultivation.

## Conflict of interest

The authors declare no conflict of interest.
